# Recognition and Assessment of Cognitive Impairment in Schizophrenia

**DOI:** 10.1192/j.eurpsy.2022.162

**Published:** 2022-09-01

**Authors:** A. Mucci, G.M. Giordano, S. Galderisi

**Affiliations:** Univeristy of Campania Luigi Vanvitelli, Department Of Psychiatry, Naples, Italy

**Keywords:** Social cognition deficits, MATRICS Consensus Cognitive Battery, Cognitive Assessment Interview, Neurocognitive impairment

## Abstract

A renewal of interest in the cognitive assessment of people with schizophrenia was related to the increasing acknowledgement of the strong relationships of cognitive deficits with functional outcome. In the early 2000’s, research focused on those aspects of cognition that demonstrated a strong correlation with a variety of functional outcome measures (community functioning, functional capacity, social skills acquisition). Later on, social cognition, which was not included in neuropsychological batteries, became also a focus as it represents a mediator of the impact of neurocognition on functioning. The renewed interest and the association with functional outcome stimulated the development of batteries specifically devoted to the cognitive assessment of subjects with schizophrenia. The MATRICS Consensus Cognitive Battery (MCCB) is the instruments with the largest evidence of good psychometric properties and strong relationship with functional outcome. The MCCB has been proposed as the gold standard in assessing cognitive impairment in subjects with schizophrenia and has been translated into 24 languages and validated in many different countries. Different instruments are also available to assess emotional processing and theory of mind which should complement MCCB and similar batteries. The long administration time limits the use of batteries in everyday clinical routine and short-administered instruments were developed as screening tools. A brief, interview-based assessment of cognitive functioning, the Cognitive Assessment Interview, has also been developed and validated for use in clinical settings or as a co-primary measure in clinical trials. The development of a guidance paper might promote the routine assessment of cognition in subjects with schizophrenia.

**Disclosure:**

Honoraria, advisory board, or consulting fees from Angelini, Astra Zeneca, Bristol-Myers Squibb, Gedeon Richter Bulgaria, Innova-Pharma, Janssen Pharmaceuticals, Lundbeck, Otsuka, Pfizer, and Pierre Fabre, for services not related to this abstract

